# The Unfolded Protein Response Is Not Necessary for the G1/S Transition, but It Is Required for Chromosome Maintenance in *Saccharomyces cerevisiae*

**DOI:** 10.1371/journal.pone.0012732

**Published:** 2010-09-14

**Authors:** Kelsey A. Henry, Heidi M. Blank, Scott A. Hoose, Michael Polymenis

**Affiliations:** Department of Biochemistry and Biophysics, Texas A&M University, College Station, Texas, United States of America; University of Washington, United States of America

## Abstract

**Background:**

The unfolded protein response (UPR) is a eukaryotic signaling pathway, from the endoplasmic reticulum (ER) to the nucleus. Protein misfolding in the ER triggers the UPR. Accumulating evidence links the UPR in diverse aspects of cellular homeostasis. The UPR responds to the overall protein synthesis capacity and metabolic fluxes of the cell. Because the coupling of metabolism with cell division governs when cells start dividing, here we examined the role of UPR signaling in the timing of initiation of cell division and cell cycle progression, in the yeast *Saccharomyces cerevisiae*.

**Methodology/Principal Findings:**

We report that cells lacking the ER-resident stress sensor Ire1p, which cannot trigger the UPR, nonetheless completed the G1/S transition on time. Furthermore, loss of UPR signaling neither affected the nutrient and growth rate dependence of the G1/S transition, nor the metabolic oscillations that yeast cells display in defined steady-state conditions. Remarkably, however, loss of UPR signaling led to hypersensitivity to genotoxic stress and a ten-fold increase in chromosome loss.

**Conclusions/Significance:**

Taken together, our results strongly suggest that UPR signaling is not necessary for the normal coupling of metabolism with cell division, but it has a role in genome maintenance. These results add to previous work that linked the UPR with cytokinesis in yeast. UPR signaling is conserved in all eukaryotes, and it malfunctions in a variety of diseases, including cancer. Therefore, our findings may be relevant to other systems, including humans.

## Introduction

In eukaryotes, proteins have to assume their native folding states, as they traffic through the endoplasmic reticulum (ER). The protein folding machinery that resides in the ER facilitates this process. Accumulation of unfolded proteins in the ER triggers the unfolded protein response (UPR). Blocking glycosylation with tunicamycin or disulfide bond formation with dithiothreitol impairs protein folding in the ER [1], [2]. In mammalian cells, ER stress is anti-mitogenic, by blocking translation of cyclin D1 and arresting the cell cycle in G1, and it also leads to apoptosis [3]. The UPR is triggered not only by acute stress, but also by physiological situations, such as altered redox status, glucose limitation, or altered protein synthesis rates [4]. For example, yeast cells growing in nitrogen-rich media are not anabolically restricted, and protein synthesis outpaces protein folding in the ER resulting in the accumulation of unfolded polypeptides [5], [6]. Furthermore, increased UPR signaling promotes cytokinesis in yeast [7].

In *S. cerevisiae*, Ire1p, an ER trans-membrane protein whose N-terminal domain is in the ER lumen, senses unfolded proteins [1]. Ire1p has kinase and endonuclease activities, which reside in separate cytosolic domains. When Ire1p dimerizes, it is auto-phosphorylated, followed by activation of Ire1p's endonuclease activity towards its only substrate, the *HAC1* mRNA [1], [8]. In the absence of UPR signaling, the *HAC1^u^* transcript is stable in the cytosol. However, an intron in the *HAC1^u^* transcript blocks efficient translation of *HAC1^u^* [1], [2]. Active Ire1p cleaves *HAC1^u^* at two splice sites removing the intron, and tRNA ligase joins the two exons, generating the *HAC1^i^* mRNA species [9], [10]. *HAC1^i^* is then translated efficiently. Hac1p^i^, together with the Gcn4p transcription factor [11], activates transcription of ∼300 UPR target genes [1], [2]. In addition, UPR signaling elevates Gcn4p levels and Gcn4p further contributes to the UPR-related transcriptional control [11]. Importantly, the *IRE1* branch of the UPR is conserved in all eukaryotes [4].

Large changes in cellular homeostasis accompany all transitions from resting to proliferative cellular states. In particular, the coupling of cellular metabolism with cell division determines the timing of the G1/S transition and the overall rate of cell proliferation [12]. Hence, the metabolic control of cell division is of great importance in the physiology of cells and organisms. Indeed metabolic changes contribute to most proliferative disorders, including cancer cell development and proliferation [13], [14]. Since the UPR is involved in numerous aspects of cellular homeostasis [4], we decided to examine the role of the UPR in linking metabolic status with cell division, using *S. cerevisiae* as a tractable system. Cells commit to a new round of cell division in late G1, before DNA synthesis in S phase, at a point called START in yeast [12]. In *S. cerevisiae*, the G1/S transition is coupled to the appearance of a bud on the cell surface, providing a convenient morphological landmark of the G1/S transition. In addition, *S. cerevisiae* is a facultative aerobe that can proliferate in steady-state continuous cultures, allowing for precise control of metabolic parameters. The above experimental properties of *S. cerevisiae* are ideal for the objective of this study, to evaluate the role of the UPR in linking cellular homeostasis with cell division. We report that the UPR has only a limited role in the integration of metabolic status with initiation of DNA replication. Remarkably, however, UPR signaling is critical for chromosome maintenance.

## Results

### UPR signaling and the G1/S transition

**Figure 1:**
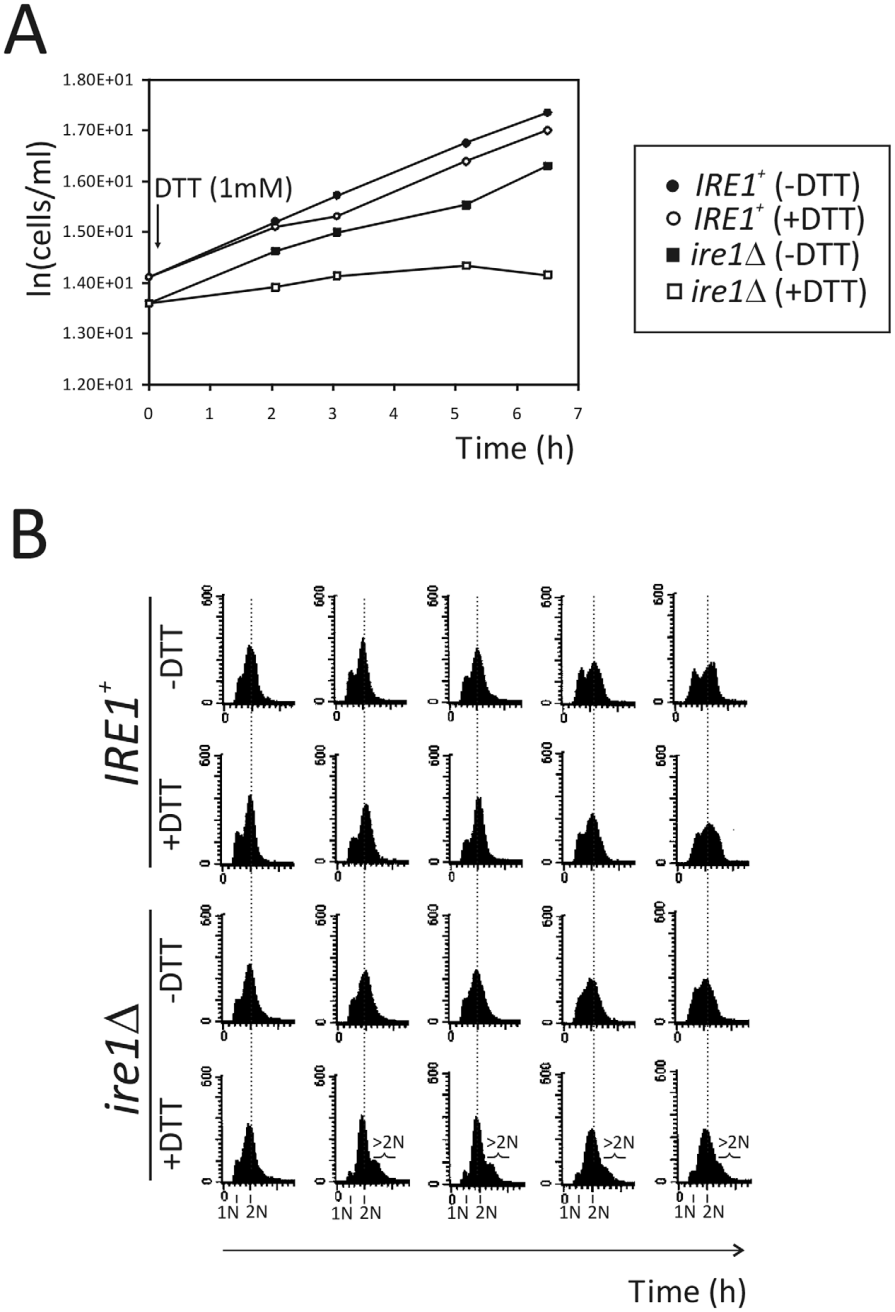
ER stress in the absence of UPR signaling blocks cell cycle progression and leads to increased ploidy. *A*, the density (in cells/ml) of *IRE1^+^* (strain X2180-5B) and *ire1Δ* (strain SCMSP176) cultures was monitored at regular intervals in the presence (or absence, as indicated) of 1 mM DTT. *B*, the DNA content of the same cultures and at the time-points shown in (A), was determined by flow cytometry. The x-axis of these histograms indicates fluorescence per cell, while the y-axis indicates the number of cells analyzed. The portion of the histograms in DTT-treated *ire1Δ* cells with increased ploidy (>2N) is indicated.

We first examined proliferation of *IRE1^+^* and *ire1Δ* cells with, or without, ER stress. We introduced ER stress by adding dithiothreitol (DTT), at 1 mM. As expected, *ire1Δ* cells proliferated very slowly after DTT addition (Fig. 1A). We also examined the DNA content of these cultures, by flow cytometry (Fig. 1B). In the presence of DTT, the majority of *ire1Δ* cells arrested with replicated DNA, while a fraction of cells had increased (>2N) ploidy. We also examined the same cells microscopically, and we noticed binucleated *ire1Δ* cells in the presence of DTT (Fig. S1), consistent with the previously reported role of the UPR in cytokinesis [7].

**Figure 2 pone-0012732-g002:**
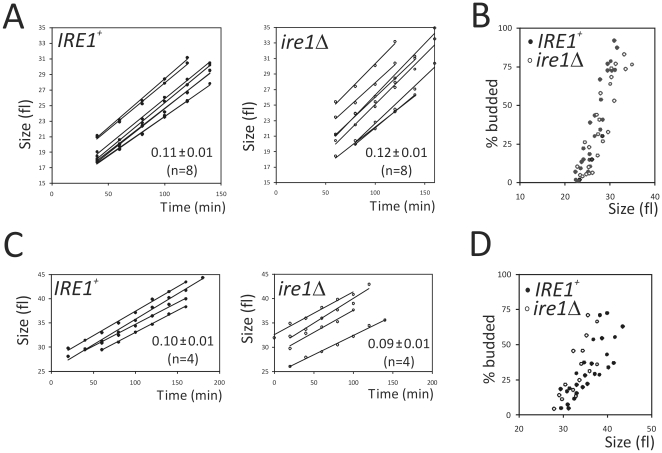
Loss of UPR signaling does not affect the timing of the G1/S transition. *A*, the rate of cell size increase for each elutriation experiment of the indicated strains is shown (they were the same strains as in Fig. 1). From these graphs, we determined the rate of size increase (shown as fl/min). The average (± SD) is shown in each case. These experiments were done in standard “rich” media with slightly lower glucose content (1% yeast extract, 2% peptone, 0.5% dextrose). *B*, from the same elutriation experiments shown in (A), we also measured the percentage of budded cells as a function of cell size (shown in fl). The data points shown were from the linear portion of each experiment, when the percentage of budded cells began to increase, and used to determine the critical size for division. *C*, and *D*, are the same type of analyses described in (A), and (B), respectively, except that the medium used was white grape juice.

We next focused on the G1/S transition. If the UPR integrates metabolic cues with cell division, then the timing of START might be altered in *ire1Δ* cells, since *ire1Δ* cells are defective in the UPR. To obtain synchronous cultures, we used centrifugal elutriation, because from a continuously growing population of cells it can separate only those cells that are in early G1, without severely altering cell growth parameters [15]. At regular intervals after elutriation, we measured cell size and the fraction of budded cells. We report two variables to compare different strains and across different experiments: the critical size for budding, at which 50% of the cells are budded, and the rate of cell size increase. Obtaining these two parameters from several repeat experiments allows for accurate estimates of G1 length. In standard laboratory media, both the rate of cell size increase (Fig. 2A), and the critical size for budding (Fig. 2B), were the same for *IRE1^+^* and *ire1Δ* cells. We also performed the same analysis in perhaps more “physiological” media, such as white grape juice [16]. Again, however, both the rate of cell size increase (Fig. 2C) and the critical size for budding (Fig. 2D), were the same for *IRE1^+^* and *ire1Δ* cells (Fig. 2B).

**Figure 3 pone-0012732-g003:**
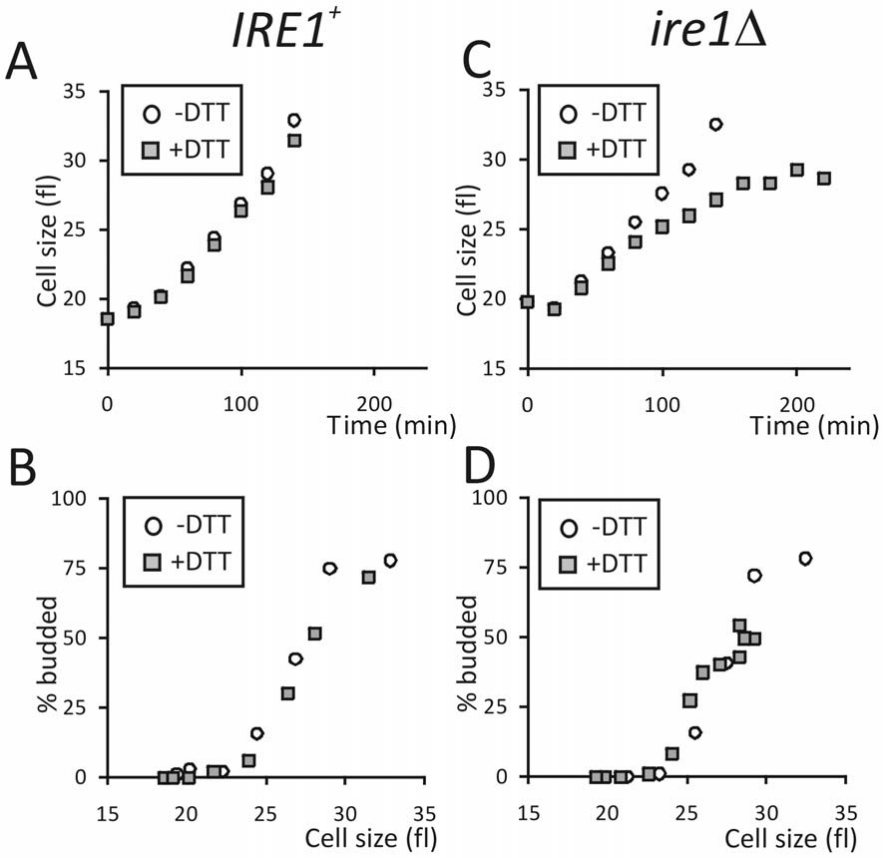
ER stress in the absence of UPR signaling decreases the rate of cell size increase, but it does not affect the critical budding size. Synchronous early-G1 cultures of *IRE1^+^* (strain X2180-5B) and *ire1Δ* (strain SCMSP176) were obtained by elutriation. Half of the elutriated culture for each strain was exposed to 1 mM DTT. The rate of cell size increase (A), and (C), and the critical budding size (B), and (D), were then monitored for *IRE1^+^*, and *ire1Δ* cells, respectively.

We then examined the timing of the G1/S transition in *IRE1^+^* and *ire1Δ* cells, but in the presence, or absence, of ER stress. For these experiments, we added DTT at 1 mM as indicated, to synchronous early-G1 cultures, immediately after elutriation. We then monitored the same parameters as in Fig. 2. It is clear that ER stress in *ire1Δ* cells lowers the rate of cell size increase (Fig. 3C), but it has no effect on the critical budding size (Fig. 3D).

**Figure 4 pone-0012732-g004:**
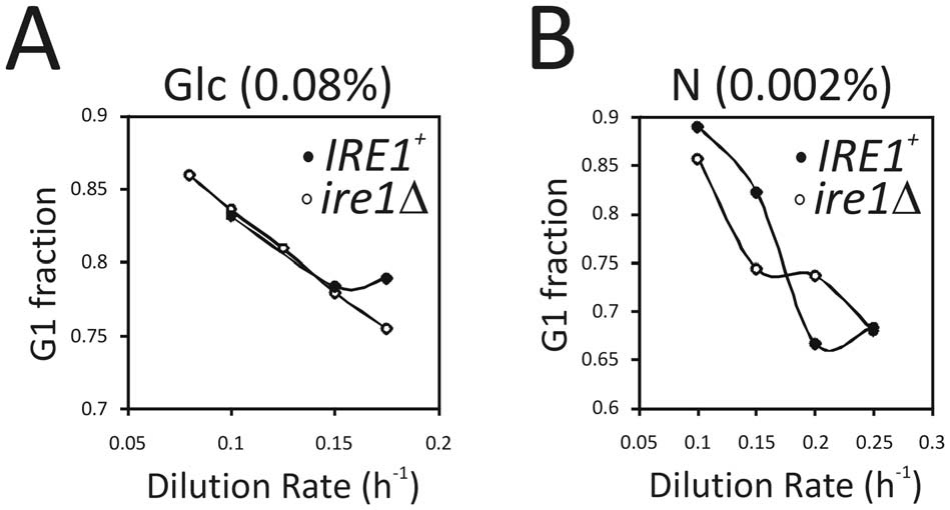
Loss of UPR signaling does not affect the nutrient or growth rate dependence of the G1/S transition. *A*, from steady-state glucose-limited (0.08% glucose) chemostat cultures of *IRE1^+^* (strain X2180-5B) or *ire1Δ* (strain SCMSP176) cells, we monitored the fraction of unbudded cells (G1 fraction), as a function of the dilution rate. *B*, a similar experiment as in (A), was done using cultures limited for nitrogen, containing 0.002% nitrogen ammonium sulfate.

To test if UPR signaling affects cell cycle progression in a nutrient or growth-rate dependent manner, we evaluated *IRE1^+^* and *ire1Δ* cells in steady-state chemostat cultures. The chemostat cultures were limited for either carbon (Fig. 4A), or nitrogen (Fig. 4B, and Fig. S3), and they were run at different dilution rates, as indicated in each case. As we lowered the dilution rate and the growth-rate declined, the fraction of cells that remained in the G1 phase of the cell cycle increased similarly in both *IRE1^+^* and *ire1Δ* cells (Fig. 4 and Fig. S3). Thus, loss of UPR signaling does not affect the nutrient and growth-rate dependence of the G1/S transition.

### UPR signaling and the yeast metabolic cycle

**Figure 5:**
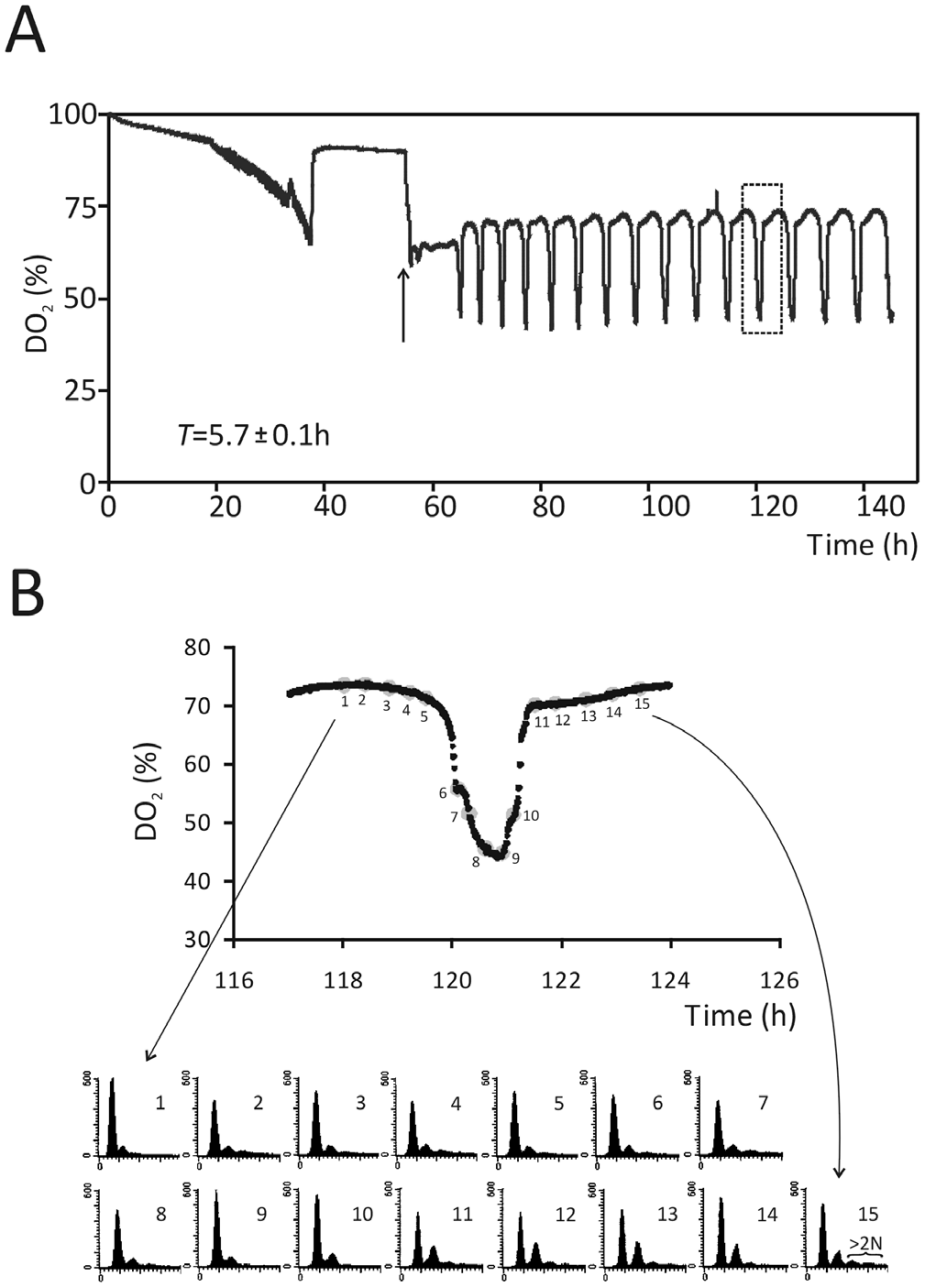
The yeast metabolic cycle of *ire1Δ* cells. *A*, oscillations of dissolved oxygen concentrations (shown as % saturation, DO_2_) in continuous cultures of *ire1Δ* cells (strain SCMSP207). The average (± SD) period, *T*, of these oscillations is indicated. The arrow indicates the point of addition of glucose-limited media (0.08% glucose) and initiation of steady-state chemostat conditions. The rectangle placed around the 120 h time point indicates a cycle that was analyzed in further detail in (B). *B*, at regular intervals as indicated, samples were taken and analyzed for DNA content by flow cytometry (shown at the bottom). The portion of the histogram with increased ploidy (>2 N) is indicated in the last time point (#15), but similar sub-populations of cells with increased ploidy were evident at other time points as well.

Under certain conditions in chemostats, yeast display periodic respiratory bursts revealing a metabolic cycle. The metabolic cycle gates cell division and cells that divide within a metabolic cycle do so synchronously [17], [18], [19], [20], [21]. Thus, during the yeast metabolic cycle the cellular metabolic and division cycles are coupled naturally. We reasoned that these metabolic oscillations are an excellent approach to evaluate whether UPR signaling couples metabolic cues with cell division. We found that *ire1Δ* cells display regular metabolic oscillations (Fig. 5A), with a period similar to *IRE1^+^* cells [17],[22],[23],[24]. We also examined the timing of DNA replication during the metabolic cycle, by monitoring the DNA content of cells during these oscillations. We found that *ire1Δ* cells initiate and complete DNA replication during the reductive phases of the metabolic cycle, when the cells do not consume oxygen (Fig. 5B). This is the expected behavior, because DNA replication during the oxidative phase is thought to damage the genome [23]. We noticed that a fraction of cells had increased (>2N) ploidy (Fig. 5B), reminiscent of the data in Fig. 1B. The increased ploidy observed during the metabolic cycle may indicate that there is some ER stress associated with the oscillations between reductive and oxidative states. Overall, however, it is clear that loss of UPR signaling does not affect the normal coupling of metabolism with cell division during the yeast metabolic cycle.

### UPR signaling and genome maintenance

**Figure 6:**
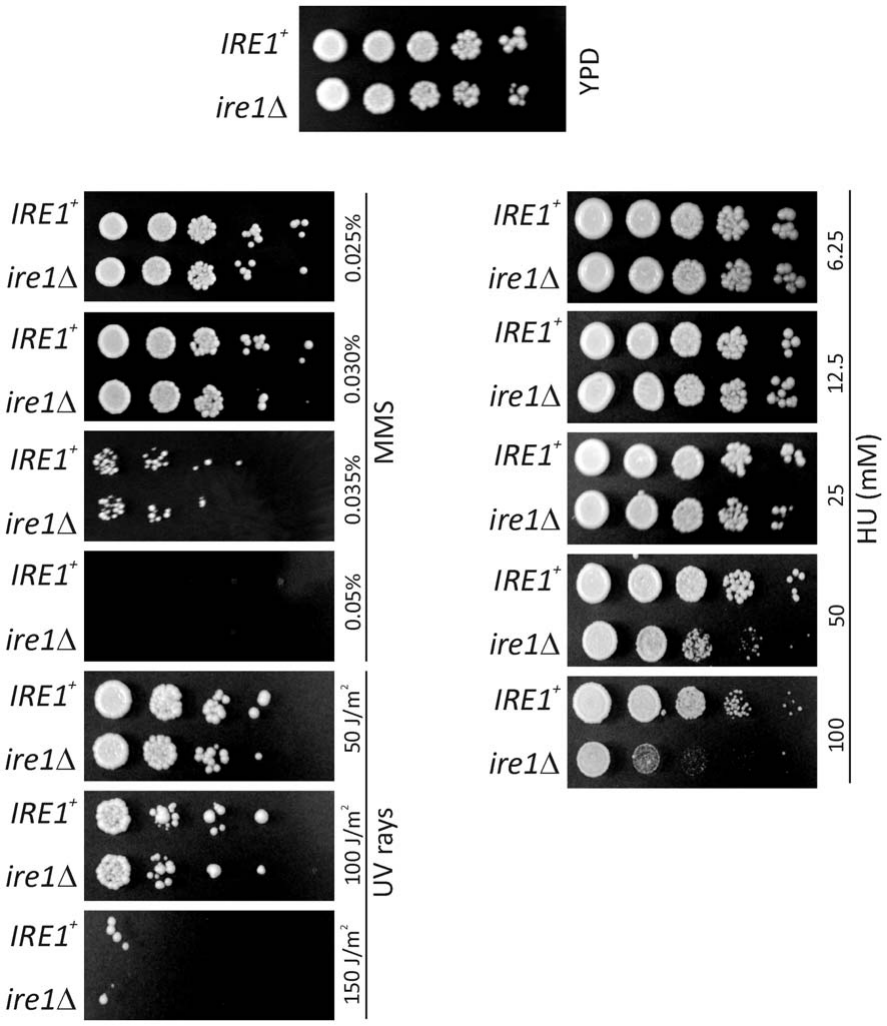
Loss of UPR signaling leads to sensitivity to hydroxyurea. *IRE1^+^* and *ire1Δ* cells (in the CEN.PK strain background) were spotted at 10-fold dilutions on YPD plates (1% yeast extract, 2% peptone, 2% dextrose), under various genotoxic conditions, as indicated. The plates were incubated at 30°C for 3 days, and photographed.

In the course of the above experiments, we noticed that after prolonged periods (>2–3 weeks) on solid media, *ire1Δ* cells lose viability earlier than their wild type counterparts do (not shown). This was surprising because exponentially growing populations of *IRE1^+^* and *ire1Δ* cells proliferate with similar rates under conditions with no obvious ER stress (see Figs. 1–5). When we exposed *ire1Δ* cells to genotoxic agents, we found that they were sensitive to high doses (>50 mM) of hydroxyurea (HU) (Fig. 6). However, loss of UPR signaling did not sensitize these cells to the alkylating agent methyl methane sulfonate (MMS) or to ultraviolet irradiation (Fig. 6). We also found that a molecular mark of checkpoint activation due to DNA damage, phosphorylation of Rad53p, accumulated normally in *ire1Δ* cells upon exposure to MMS (Fig. S4). Finally, *ire1Δ* cells were not more resistant to cycloheximide (Fig. S5), arguing that their background mutation rate is not altered.

**Figure 7:**
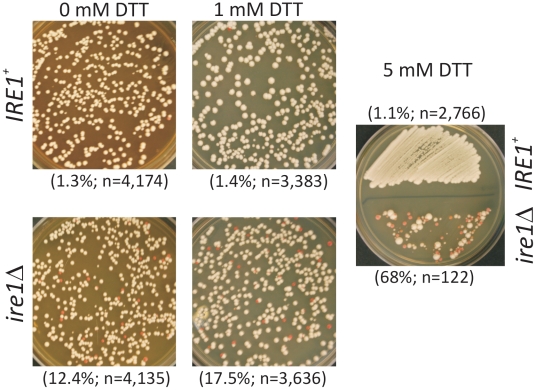
Loss of UPR signaling leads to chromosome loss. Sectoring assay for chromosome loss, with *IRE1^+^* or *ire1Δ* cells, in the YPH363 strain background. Formation of red sectors indicates chromosome loss. Representative plates for each strain are shown, at 0 mm and 1 mM DTT. There were very few viable *ire1Δ* cells in the presence of 5 mM DTT, and this is illustrated by a streak of the two strains on the same plate containing 5 mM DTT. The total number of colonies counted and the percentage of sectored colonies in each case are shown.

We then examined the mitotic fidelity of chromosome transmission in *ire1Δ* cells, using a well-established colony sectoring assay [25]. This assay uses the red pigmentation of yeast *ade2* mutants to score chromosome loss. The strain used carries the ochre allele *ade2-101*, and a non-essential chromosome carrying the ochre suppressor tRNA *SUP11*. Thus, as long as the *SUP11*-carrying chromosome is present, the cells appear white. On the other hand, if the marker chromosome is lost, colonies form red sectors. We found that the rate of chromosome loss is significantly elevated (∼10-fold) in *ire1Δ* cells (Fig. 7). ER stress, induced by the addition of 1 mM DTT, exacerbated the elevated rate of chromosome loss in *ire1Δ* cells. Although this exacerbation of the chromosome loss is significant (*P*<0.0001, based on a χ^2^ test), it should be noted that the cells are under severe stress, with a non-functional UPR. At higher doses (5 mM) of DTT, when the viability of *ire1Δ* cells dropped dramatically, the few survivors usually displayed chromosome loss (Fig. 7). Finally, we noted that the strain used in the chromosome loss assay is not sensitive to hydroxyurea (Fig. S6A), but hydroxyurea increased the rate of chromosome loss of *ire1Δ* cells (Fig. S6B). Overall, it clear that UPR signaling is required for maintaining the transmission of chromosomes with high fidelity.

## Discussion

A role for the secretory pathway in cell division is almost self-evident in *S. cerevisiae*, since bud formation and cell surface growth depend on secretory processes. Pioneering studies of the secretory pathway established that cell division is blocked at the non-permissive temperature of conditional secretion-defective mutants of *S. cerevisiae* [26]. Originally, the UPR was perceived as a stress response, but it has an increasingly recognized role in cellular homeostasis, especially in vertebrates [4]. Specifically for *S. cerevisiae*, a recent study revealed a function for the UPR during cytokinesis [7]. Importantly, however, that study did not evaluate events in the cell cycle prior to DNA replication [7]. Since the G1/S transition is very sensitive to changes in cellular homeostasis and metabolic status [12], we decided to examine in detail the role of UPR signaling during the G1/S transition.

ER stress is obviously anti-mitogenic in the absence of UPR signaling (Fig. 1), because cells grow in size much slower (Fig. 3A and 3C). In contrast, secretory perturbations do not alter at all the critical size requirement for division (Fig. 3B and 3D). These findings are consistent with the original reports of conditional secretory mutants, where a defect in cell surface growth was noted [26]. Our findings also strongly indicate that the UPR does not set the metabolic/cell size requirement for cell division, but in the presence of ER stress, the UPR is required to ensure that these growth requirements are met before division.

In the absence of ER stress, however, the timing of initiation of cell division was unaffected, whether the UPR was functional, or not. We reached this conclusion from synchronous cultures in laboratory media and grape juice cultures (Fig. 2). Grape juice is very rich in carbon, but poor in nitrogen [16]. We also performed a series of chemostat experiments to separate further nutrient and growth-rate variables. In all conditions tested, *IRE1^+^* cells behaved similarly to *ire1Δ* cells (Fig 4). Similar approaches have been used widely to probe cellular physiology [27],[28],[29]. Finally, we employed approaches that reveal a metabolic cycle in yeast cultures. This procedure has many advantages. For example, cell division and metabolism are coupled naturally. In every metabolic cycle, one can observe an oxidative, an early reductive, and a late reductive phase. In contrast, in batch cultures there are no discernible metabolic landmarks. The metabolic cycle was shown recently to be a cell-autonomous property of yeast cells, observed in a variety of conditions [30]. Therefore, metabolic oscillations probably reflect a general, intrinsic biological property of yeast cells. We found that loss of UPR signaling does not affect the yeast metabolic cycle (Fig. 5). Overall, we used a diverse array of approaches to probe the role of the UPR in G1/S transition. Although the UPR may be triggered by changes in nutrient status [4], our data suggest that it is unlikely that the UPR plays a major role in the integration of metabolic cues to affect the G1/S transition in yeast.

We then discovered that loss of *IRE1* leads to chromosome loss (Fig. 7). While the Ire1 pathway has been involved in cell survival pathways in vertebrates [4], to our knowledge, a role for Ire1 in chromosome maintenance has not been reported in any system. However, from a large-scale genetic study in yeast, *HAC1* was among the genes affecting chromosome stability in a dosage-dependent manner [31]. Our results implicating *IRE1* in the same process are consistent with that study. Furthermore, in animals, the Hac1p ortholog XBP1 might up-regulate expression of genes involved in DNA damage and repair pathways [32]. Again, this is of potential relevance to this study, given the remarkable conservation between XBP1 and Hac1p. In fact, the *XBP1^u^* and *HAC1^u^* transcripts are processed in the same way by the mammalian IRE1 and yeast Ire1p, respectively [4],[33]. Although we found that *ire1Δ* cells are hypersensitive to hydroxyurea (Fig. 6), *ire1Δ* cells were not hypersensitive to other genotoxic treatments, such as alkylating agents, or ultraviolet radiation (Fig. 6). Hydroxyurea does not directly damage DNA, but it arrests DNA chain elongation synthesis [34], which then leads to genomic instability. Perhaps the UPR has specific, and not generic, roles in genome maintenance pathways. It is also possible that hydroxyurea somehow causes ER stress. Overall, the results we report in this study add to the multiple roles of the UPR in cell physiology. Further clarification of the role of the UPR in chromosome maintenance will require a detailed molecular understanding of the downstream factors(s) involved. Because of the high degree of conservation of the Ire1 branch in all eukaryotes, our findings may be relevant to other systems.

## Materials and Methods

### Strains

The reference strains used in this study were the haploid wild type strains X2180-5B (*MAT*a *SUC2 mal mel gal2 CUP1*, a gift from Dr. Robert Mortimer, Yeast Genetic Stock Center); CEN.PK (*MAT*α, a gift from Dr. Ben Tu, UT Southwestern Medical School [24]); and a strain engineered for chromosome loss assays, YPH363 (MATα *ura3-52 lys2-801 leu2-Δ1 ade2-101 his3-Δ200 [CFIII(CEN3.L) CFVII(RAD2.d) URA3 SUP11]*, a gift from Dr. Phil Hieter, Univ. of British Columbia [25]). In each of these strains, we replaced the *IRE1^+^* locus with an *ire1Δ::KANMX* cassette, following a standard, single-step PCR replacement protocol, as described [35]. The transformants were genotyped for the presence of the *ire1Δ::KANMX* cassette and the absence of *IRE1^+^*, to obtain strains SCMSP176 (*ire1Δ::KANMX* in the X2180-5B background), SCMSP207 (*ire1Δ::KANMX* in the CEN.PK background), and SCMSP210 (*ire1Δ::KANMX* in the YPH363 background).

### Yeast cultivation and cell cycle analysis

For batch cultures we followed standard protocols [36]. Centrifugal elutriation conditions, DNA content, “growth rate” and “critical size” analyses were done as we have described [22],[37]. For the elutriation experiments in Fig. 2B, we used grape juice (Welch's® 100% white grape juice from Niagara grapes). For the chemostat experiments shown in Fig. 4A, the cultures contained 1.7 g l^−1^ yeast nitrogen base (Difco, MI), without ammonium sulfate and amino acids, 0.8 g l^−1^ dextrose, 5 g l^−1^ ammonium sulfate. For the chemostat experiments shown in Fig. 4B, the cultures contained 1.7 g l^−1^ yeast nitrogen base, without ammonium sulfate and amino acids, 20 g l^−1^ dextrose, 0.02 g l^−1^ ammonium sulfate. For the chemostat experiments shown in Figure S2, we also examine different concentrations of nitrogen, as indicated. Standard batch cultures in synthetic media contain 0.5% ammonium sulfate [36]. We tested several nitrogen concentrations and we found that cultures containing anywhere from 0.05% to 0.000847% are limiting for growth, but to different degrees (Figure S2A). We [38] and others [39] [40] have used previously chemostat cultures containing 0.05% ammonium sulfate to examine nitrogen limitation. Under these conditions, the fraction of cells that remain in the G1 phase of the cell cycle does not change as a function of growth rate (see Figure S2B, and [38]). However, at lower nitrogen concentrations there is a gradual increase in the fraction of cells that remain in G1, as a function of growth-rate (see Figure S2B, and [28]). For this reason, we decided to use media containing 0.002% nitrogen in the experiment we show in Fig. 4B, to ensure that any differences between *IRE1^+^* and *ire1Δ* cells will be evident.

Conditions for yeast metabolic oscillations in chemostat cultures were identical to previously published protocols [17].

All chemicals were from Sigma (St. Louis, MO), unless specified otherwise.

### Rad53p phosphorylation

Whole cell extracts were prepared by beating the cells with glass beads in SDS-PAGE buffer [41]. Samples were heated and centrifuged prior to loading on an Invitrogen (Carlsbad, CA) NuPage(R) Novex 4–12% Bis-Tris 1.0 mm 15-well mini-gel, and samples were electrophoresed at 75 V for ∼5 h at 4°C. The gel was blotted onto PVDF membrane using the Invitrogen iBlot(R) Dry Blotting System. Rad53p was detected with an anti-Rad53p antibody (Santa-Cruz Biotechnology (Santa Cruz, CA); sc-6749), according to their recommendations. The blot was developed using SuperSignal West Pico Chemiluminescent Substrate from Thermo Scientific (Waltham, MA) and imaged on a BioRad (Hercules, CA) ChemiDoc XRS system.

## Supporting Information

Figure S1 ER stress in the absence of UPR signaling leads to multinucleated cells. From the same experiment shown in Figure 1 and at the 2 hr time-point, in the absence (A) or presence (B) of 1 mM DTT, we stained *IRE1^+^* and *ire1Δ* cells with 4′,6-diamidino-2-phenylindole (DAPI), to visualize their nuclear morphology. The cells were photographed through phase optics and by fluorescence microscopy, and the two images were overlaid. The arrows indicate binucleated *ire1Δ* cells in the presence of 1 mM DTT.
(8.58 MB TIF)

Figure S2 Cell cycle progression and nitrogen limitation. A, the cell density of *IRE1^+^* batch cultures (strain X2180-5B) after 4 days in minimal media containing the indicated amounts of ammonium sulfate is shown. B, from steady-state chemostat cultures of *IRE1^+^* (strain X2180-5B) cells containing the indicated amounts of nitrogen, we monitored the fraction of unbudded cells (G1 fraction), as a function of the dilution rate.
(7.24 MB TIF)

Figure S3 Loss of UPR signaling in the CEN.PK strain background does not affect the growth rate dependence of the G1/S transition under nitrogen limitation. *IRE1^+^* and *ire1Δ* cells (in the CEN.PK strain background) were cultivated in nitrogen-limited chemostats, with media containing 0.002% ammonium sulfate. The cultures were sampled at several different dilution rates, as indicated, and the DNA content was determined by flow cytometry, as described in Fig. 1. The percentage of cells with G1 DNA content is shown in each case.
(4.05 MB TIF)

Figure S4 Phosphorylation of Rad53p accumulates normally in *ire1Δ* cells upon exposure to MMS. Exponentially growing *IRE1^+^* and *ire1Δ* liquid cultures (both in the CEN.PK background) were split in half and exposed to 0.1% MMS as indicated. At the indicated time-points after MMS exposure, samples were collected for SDS-PAGE and immunobloting, against Rad53p. Phosphorylated Rad53p migrates slower than the unphosphorylated form, as indicated by the arrows (top). The same blot was stained with Coomassie to indicate loading (bottom).
(5.69 MB TIF)

Figure S5 Loss of *IRE1^+^* does not increase resistance to cycloheximide. *IRE1^+^* or *ire1Δ* cells, in the YPH363 strain background, were plated on solid media in the absence or presence of 2.5 µg/ml cycloheximide, as indicated. The plates were then incubated at 30°C for 4 days.
(5.86 MB TIF)

Figure S6 Hydroxyurea increases the rate of chromosome loss of *ire1Δ* cells. A, *ire1Δ*, but not *IRE1^+^*, cells in the YPH363 strain background are sensitive to hydroxyurea. Cells were spotted at 10-fold dilutions on YPD plates (1% yeast extract, 2% peptone, 2% dextrose), containing hydroxyurea, as indicated. The plates were incubated at 30°C for 3 days, and photographed. B, Sectoring assay for chromosome loss, with *IRE1^+^* or *ire1Δ* cells, in the YPH363 strain background, in the presence of 50 mM hydroxyurea. The assay was done as we described in Fig. 7.
(9.52 MB TIF)

## References

[1] Patil C, Walter P (2001). Intracellular signaling from the endoplasmic reticulum to the nucleus: the unfolded protein response in yeast and mammals.. Curr Opin Cell Biol.

[2] Rutkowski DT, Kaufman RJ (2004). A trip to the ER: coping with stress.. Trends Cell Biol.

[3] Zhao L, Ackerman SL (2006). Endoplasmic reticulum stress in health and disease.. Curr Opin Cell Biol.

[4] Rutkowski DT, Hegde RS (2010). Regulation of basal cellular physiology by the homeostatic unfolded protein response.. J Cell Biol.

[5] Kaufman RJ, Scheuner D, Schroder M, Shen X, Lee K (2002). The unfolded protein response in nutrient sensing and differentiation.. Nat Rev Mol Cell Biol.

[6] Schroder M, Chang JS, Kaufman RJ (2000). The unfolded protein response represses nitrogen-starvation induced developmental differentiation in yeast.. Genes Dev.

[7] Bicknell AA, Babour A, Federovitch CM, Niwa M (2007). A novel role in cytokinesis reveals a housekeeping function for the unfolded protein response.. J Cell Biol.

[8] Niwa M, Patil CK, DeRisi J, Walter P (2005). Genome-scale approaches for discovering novel nonconventional splicing substrates of the Ire1 nuclease.. Genome Biol.

[9] Sidrauski C, Cox JS, Walter P (1996). tRNA ligase is required for regulated mRNA splicing in the unfolded protein response.. Cell.

[10] Sidrauski C, Walter P (1997). The transmembrane kinase Ire1p is a site-specific endonuclease that initiates mRNA splicing in the unfolded protein response.. Cell.

[11] Patil CK, Li H, Walter P (2004). Gcn4p and novel upstream activating sequences regulate targets of the unfolded protein response.. PLoS Biol.

[12] Pringle JR, Hartwell LH, Strathern JD, Jones EW, Broach JR (1981). The *Saccharomyces cerevisiae* cell cycle.. The molecular biology of the yeast *Saccharomyces*.

[13] Vander Heiden MG, Cantley LC, Thompson CB (2009). Understanding the Warburg effect: the metabolic requirements of cell proliferation.. Science.

[14] Hsu PP, Sabatini DM (2008). Cancer cell metabolism: Warburg and beyond.. Cell.

[15] Creanor J, Mitchison J (1979). Reduction of perturbations in leucine incorporation in synchronous cultures of *Schizosaccharomyces pombe* made by elutriaton.. J Gen Microbiol.

[16] Delneri D, Hoyle DC, Gkargkas K, Cross EJ, Rash B (2008). Identification and characterization of high-flux-control genes of yeast through competition analyses in continuous cultures.. Nat Genet.

[17] Tu BP, Kudlicki A, Rowicka M, McKnight SL (2005). Logic of the yeast metabolic cycle: temporal compartmentalization of cellular processes.. Science.

[18] Porro D, Martegani E, Ranzi BM, Alberghina L (1988). Oscillations in continuous cultures of yeast: A segragated parameter analysis.. Biotechnol Bioengin.

[19] Klevecz RR, Bolen J, Forrest G, Murray DB (2004). A genomewide oscillation in transcription gates DNA replication and cell cycle.. Proc Natl Acad Sci U S A.

[20] Murray DB, Beckmann M, Kitano H (2007). Regulation of yeast oscillatory dynamics.. Proc Natl Acad Sci U S A.

[21] Murray DB, Klevecz RR, Lloyd D (2003). Generation and maintenance of synchrony in Saccharomyces cerevisiae continuous culture.. Exp Cell Res.

[22] Blank HM, Gajjar S, Belyanin A, Polymenis M (2009). Sulfur metabolism actively promotes initiation of cell division in yeast.. PLoS ONE.

[23] Chen Z, Odstrcil EA, Tu BP, McKnight SL (2007). Restriction of DNA replication to the reductive phase of the metabolic cycle protects genome integrity.. Science.

[24] Tu BP, Mohler RE, Liu JC, Dombek KM, Young ET (2007). Cyclic changes in metabolic state during the life of a yeast cell.. Proc Natl Acad Sci U S A.

[25] Spencer F, Gerring SL, Connelly C, Hieter P (1990). Mitotic chromosome transmission fidelity mutants in Saccharomyces cerevisiae.. Genetics.

[26] Novick P, Schekman R (1979). Secretion and cell-surface growth are blocked in a temperature-sensitive mutant of Saccharomyces cerevisiae.. Proc Natl Acad Sci U S A.

[27] Castrillo JI, Zeef LA, Hoyle DC, Zhang N, Hayes A (2007). Growth control of the eukaryote cell: a systems biology study in yeast.. J Biol.

[28] Brauer MJ, Huttenhower C, Airoldi EM, Rosenstein R, Matese JC (2008). Coordination of growth rate, cell cycle, stress response, and metabolic activity in yeast.. Mol Biol Cell.

[29] Saldanha AJ, Brauer MJ, Botstein D (2004). Nutritional homeostasis in batch and steady-state culture of yeast.. Mol Biol Cell.

[30] Silverman SJ, Petti AA, Slavov N, Parsons L, Briehof R Metabolic cycling in single yeast cells from unsynchronized steady-state populations limited on glucose or phosphate.. Proc Natl Acad Sci U S A.

[31] Strome ED, Wu X, Kimmel M, Plon SE (2008). Heterozygous screen in Saccharomyces cerevisiae identifies dosage-sensitive genes that affect chromosome stability.. Genetics.

[32] Acosta-Alvear D, Zhou Y, Blais A, Tsikitis M, Lents NH (2007). XBP1 controls diverse cell type- and condition-specific transcriptional regulatory networks.. Mol Cell.

[33] Wiseman RL, Haynes CM, Ron D (2010). SnapShot: The unfolded protein response.. Cell.

[34] Koc A, Wheeler LJ, Mathews CK, Merrill GF (2004). Hydroxyurea arrests DNA replication by a mechanism that preserves basal dNTP pools.. J Biol Chem.

[35] Longtine MS, McKenzie A, Demarini DJ, Shah NG, Wach A (1998). Additional modules for versatile and economical PCR-based gene deletion and modification in *Saccharomyces cerevisiae*.. Yeast.

[36] Kaiser C, Michaelis S, Mitchell A (1994). Methods in Yeast Genetics..

[37] Blank HM, Li C, Mueller JE, Bogomolnaya LM, Bryk M (2008). An increase in mitochondrial DNA promotes nuclear DNA replication in yeast.. PLoS Genet.

[38] Guo J, Bryan BA, Polymenis M (2004). Nutrient-specific effects in the coordination of cell growth with cell division in continuous cultures of *Saccharomyces cerevisiae*.. Arch Microbiol.

[39] Baganz F, Hayes A, Farquhar R, Butler PR, Gardner DC (1998). Quantitative analysis of yeast gene function using competition experiments in continuous culture.. Yeast.

[40] Baganz F, Hayes A, Marren D, Gardner DC, Oliver SG (1997). Suitability of replacement markers for functional analysis studies in Saccharomyces cerevisiae.. Yeast.

[41] Laemmli UK (1970). Cleavage of structural proteins during the assembly of the head of bacteriophage T4.. Nature.

