# Supplementary figures and images for: The Unfolded Protein Response Is Not Necessary for the G1/S Transition, but It Is Required for Chromosome Maintenance in Saccharomyces cerevisiae
Source: PLoS One. 2010 Sep 14;5(9):e12732. doi: 10.1371/journal.pone.0012732 (PMC2939067; doi:10.1371/journal.pone.0012732)

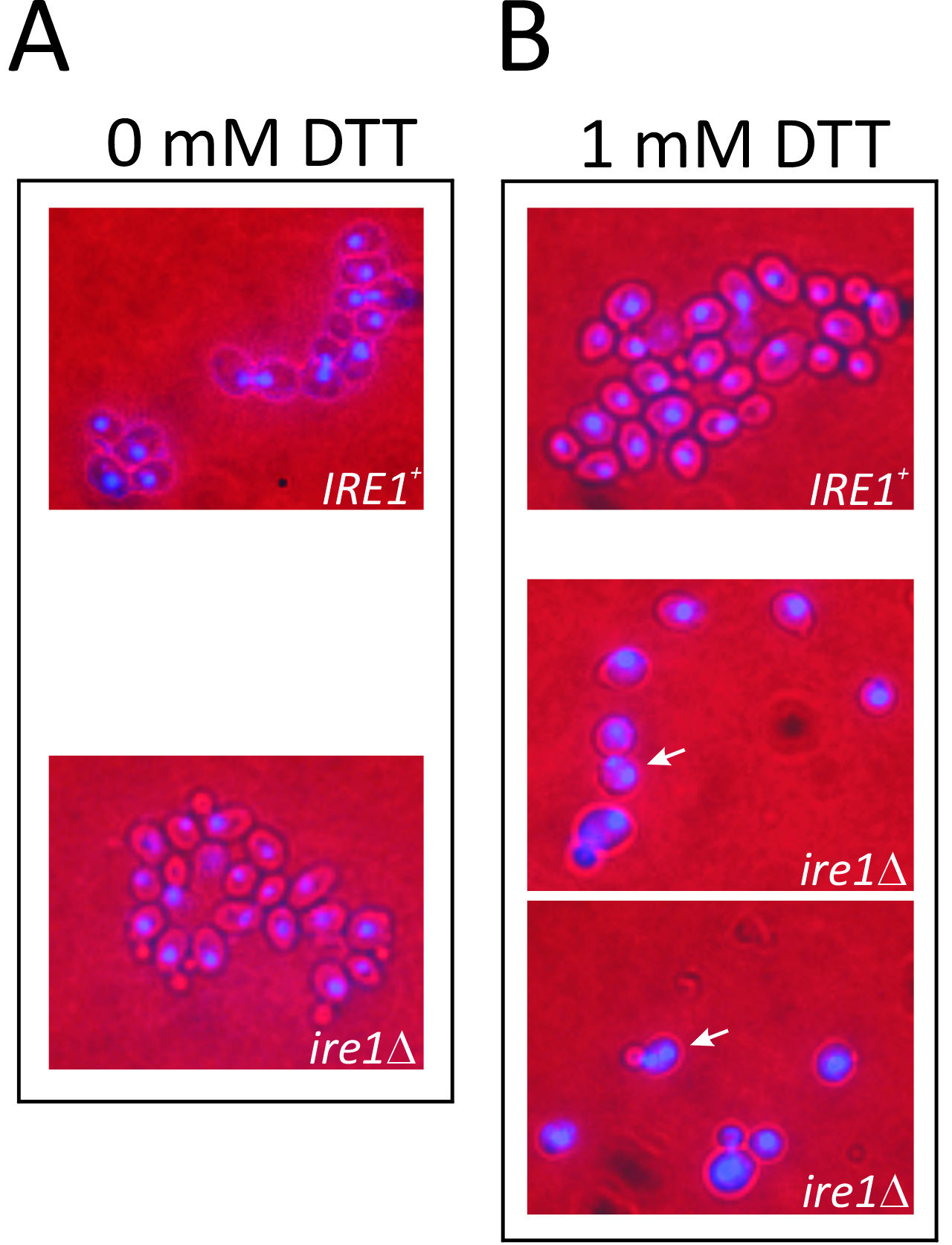

Supplement: Figure S1 — ER stress in the absence of UPR signaling leads to multinucleated cells. From the same experiment shown in Figure 1 and at the 2 hr time-point, in the absence (A) or presence (B) of 1 mM DTT, we stained IRE1+ and ire1Δ cells with 4′,6-diamidino-2-phenylindole (DAPI), to visualize their nuclear morphology. The cells were photographed through phase optics and by fluorescence microscopy, and the two images were overlaid. The arrows indicate binucleated ire1Δ cells in the presence of 1 mM DTT. (8.58 MB TIF) [file pone.0012732.s001.tif]

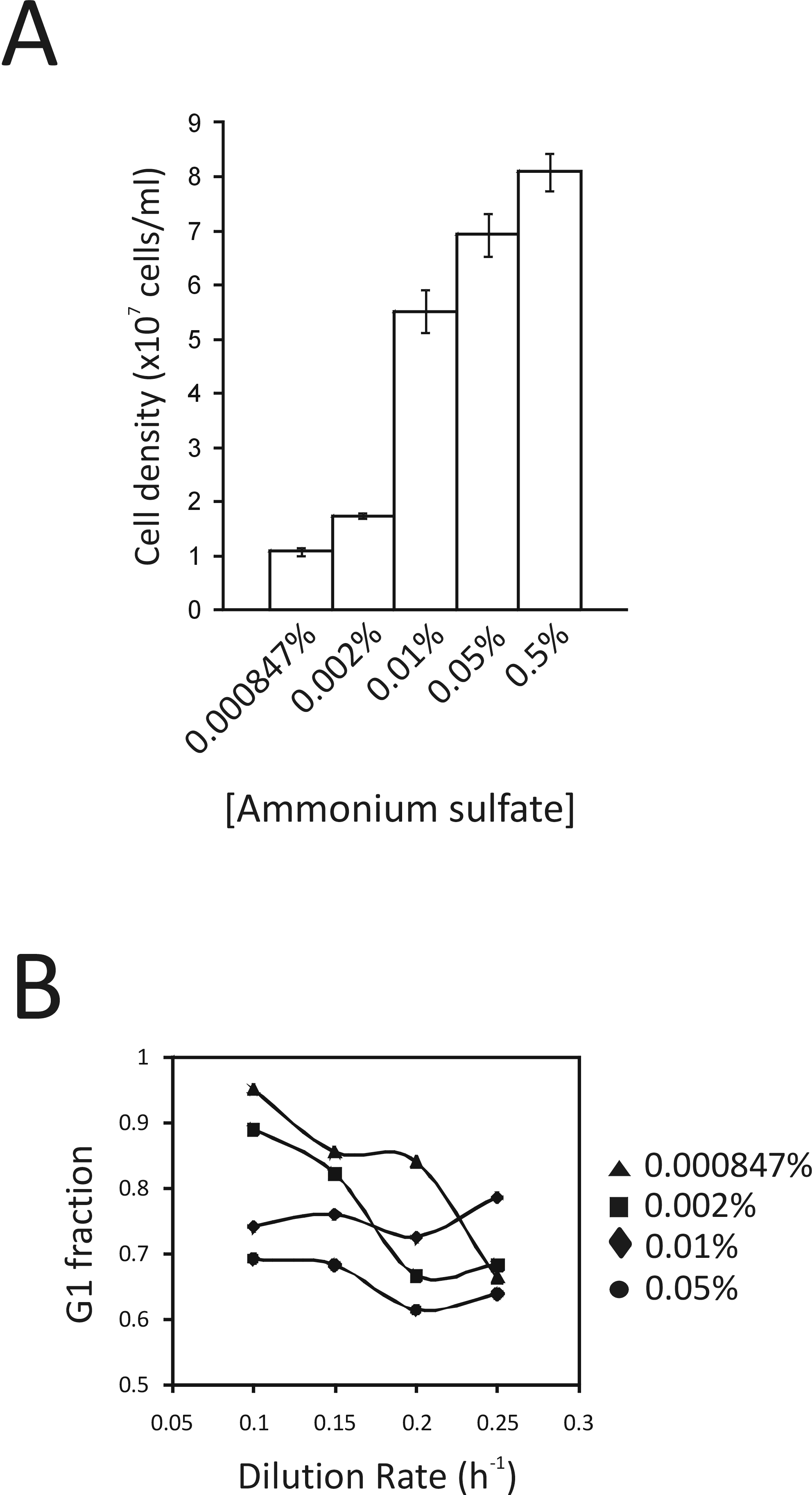

Supplement: Figure S2 — Cell cycle progression and nitrogen limitation. A, the cell density of IRE1+ batch cultures (strain X2180-5B) after 4 days in minimal media containing the indicated amounts of ammonium sulfate is shown. B, from steady-state chemostat cultures of IRE1+ (strain X2180-5B) cells containing the indicated amounts of nitrogen, we monitored the fraction of unbudded cells (G1 fraction), as a function of the dilution rate. (7.24 MB TIF) [file pone.0012732.s002.tif]

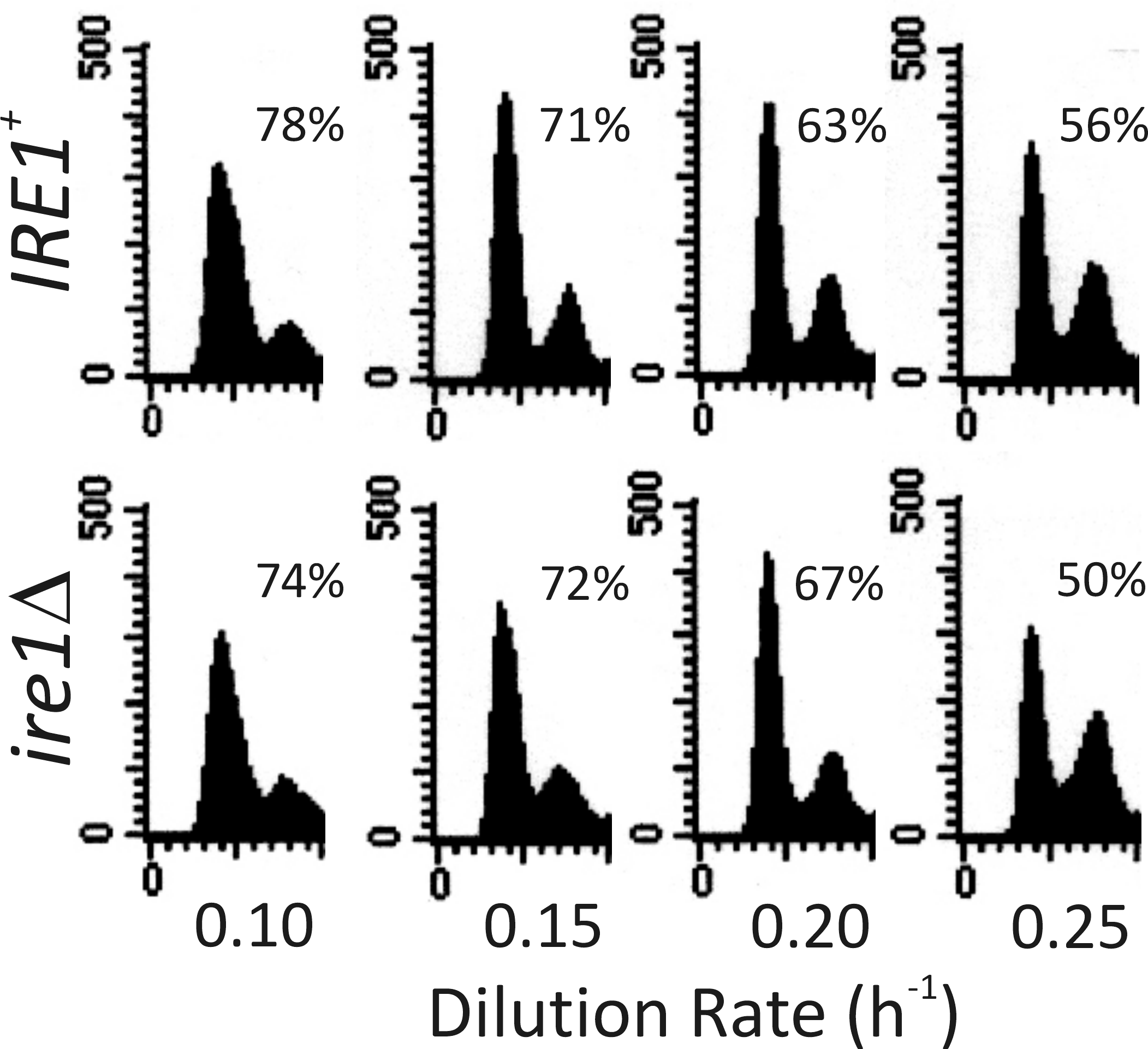

Supplement: Figure S3 — Loss of UPR signaling in the CEN.PK strain background does not affect the growth rate dependence of the G1/S transition under nitrogen limitation. IRE1+ and ire1Δ cells (in the CEN.PK strain background) were cultivated in nitrogen-limited chemostats, with media containing 0.002% ammonium sulfate. The cultures were sampled at several different dilution rates, as indicated, and the DNA content was determined by flow cytometry, as described in Fig. 1. The percentage of cells with G1 DNA content is shown in each case. (4.05 MB TIF) [file pone.0012732.s003.tif]

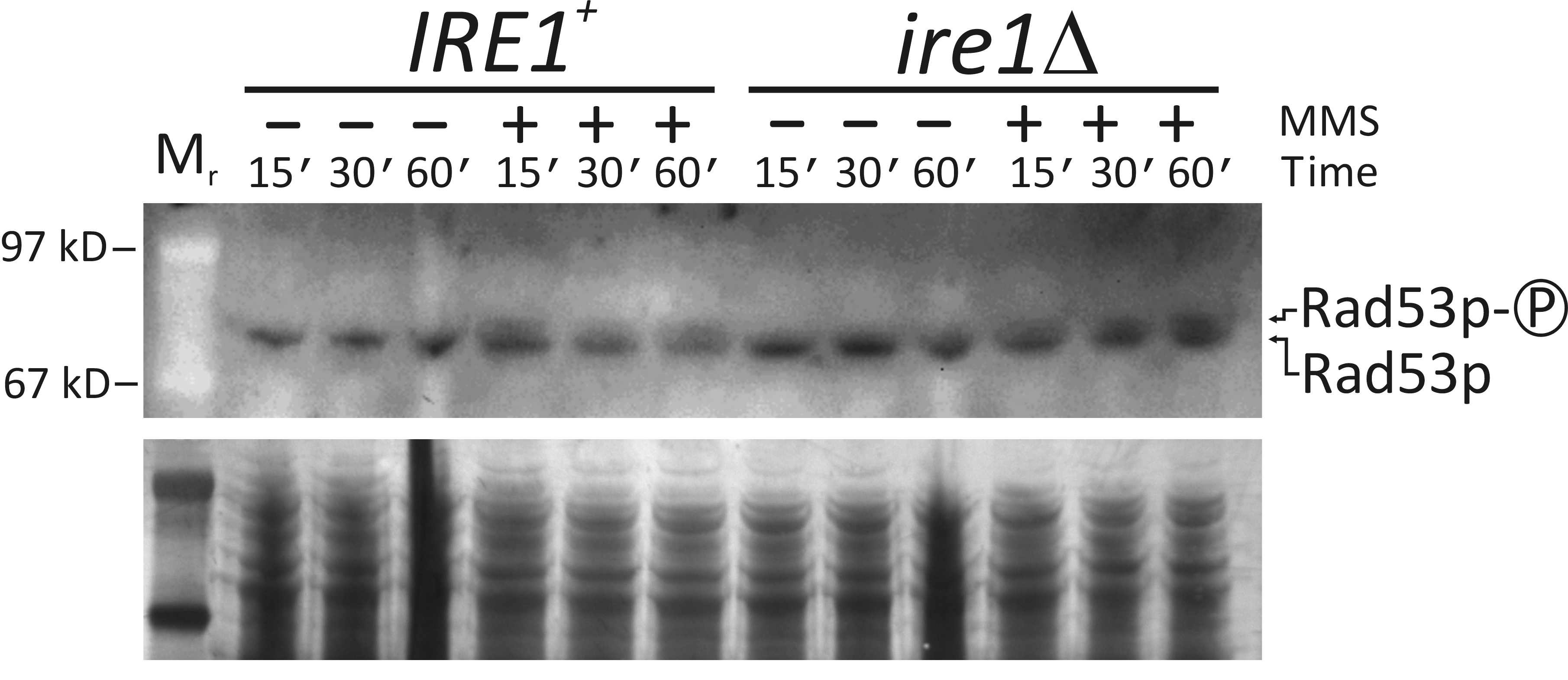

Supplement: Figure S4 — Phosphorylation of Rad53p accumulates normally in ire1Δ cells upon exposure to MMS. Exponentially growing IRE1+ and ire1Δ liquid cultures (both in the CEN.PK background) were split in half and exposed to 0.1% MMS as indicated. At the indicated time-points after MMS exposure, samples were collected for SDS-PAGE and immunobloting, against Rad53p. Phosphorylated Rad53p migrates slower than the unphosphorylated form, as indicated by the arrows (top). The same blot was stained with Coomassie to indicate loading (bottom). (5.69 MB TIF) [file pone.0012732.s004.tif]

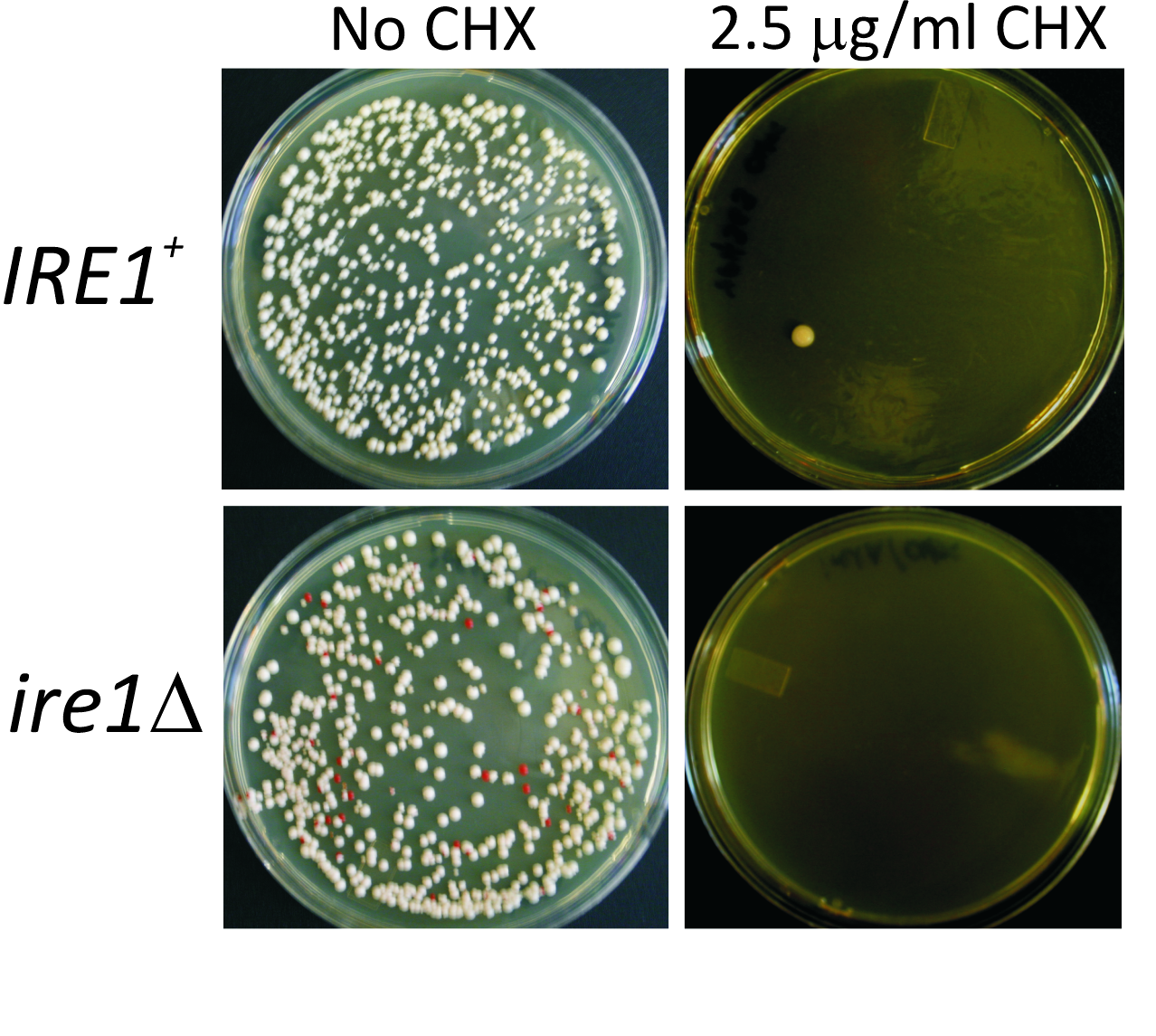

Supplement: Figure S5 — Loss of IRE1+ does not increase resistance to cycloheximide. IRE1+ or ire1Δ cells, in the YPH363 strain background, were plated on solid media in the absence or presence of 2.5 µg/ml cycloheximide, as indicated. The plates were then incubated at 30°C for 4 days. (5.86 MB TIF) [file pone.0012732.s005.tif]

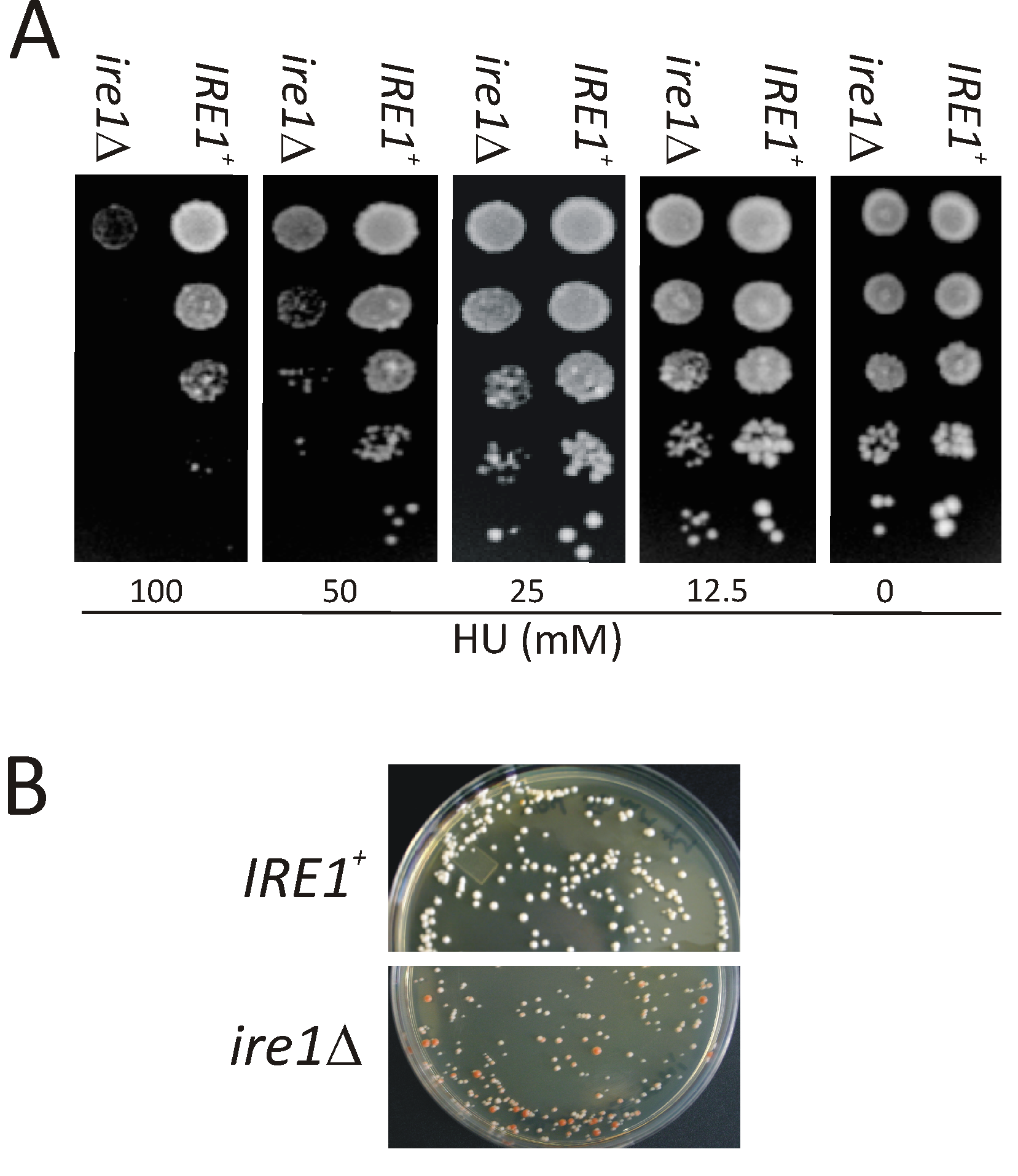

Supplement: Figure S6 — Hydroxyurea increases the rate of chromosome loss of ire1Δ cells. A, ire1Δ, but not IRE1+, cells in the YPH363 strain background are sensitive to hydroxyurea. Cells were spotted at 10-fold dilutions on YPD plates (1% yeast extract, 2% peptone, 2% dextrose), containing hydroxyurea, as indicated. The plates were incubated at 30°C for 3 days, and photographed. B, Sectoring assay for chromosome loss, with IRE1+ or ire1Δ cells, in the YPH363 strain background, in the presence of 50 mM hydroxyurea. The assay was done as we described in Fig. 7. (9.52 MB TIF) [file pone.0012732.s006.tif]
